# Supplementary material for: A new record of kelp Lessonia spicata (Suhr) Santelices in the Sub-Antarctic Channels: implications for the conservation of the “huiro negro” in the Chilean coast
Source: PeerJ. 2019 Sep 19;7:e7610. doi: 10.7717/peerj.7610 (PMC6754978; doi:10.7717/peerj.7610)
Supplement: Supplemental Information 2 [file peerj-07-7610-s002.docx]

**PHYLOGENETIC ANALYSIS**

**ITS gene**

>LesA [organism=Lessonia spicata] [country=Chile: Castillo channel, Torpedo Island, Katalalixar National Reserve] internal transcribed spacer 1, partial sequence

ACCCCCCCGCTCTACAAATTGTCTGTGACGT-CGCCGTGGAAACTCCCTCGGAGGCGAGCGA-GCGATCGTCTAA---ACCCCGAGAAAGTGAAGCCGTTATGCGAAGTTGGGCGAGGGGCGCCTCGCCGAGGGTTTTTCTTTTCCCTTTCCCCTTCTTGTCTC-TTTTCTTCCCTTTTCGGGGATTTACGGGACGGGACGGGAAGGGTGAAGGAAGCTCTCGAACCAAAGCGCACCCCACTTTTCAACCCCATTAAACTCTGAATCTGAACTC-AAAGGGGGGCAGCGCTGTGCCGCGGCTCCCCCAACCTTTAACGTTGTAAAACTTTCAGCGACGGATGTCTTGGCTCCCACAACGATGAAGAACGCAGCGAAATGCGATACGTCTTGCGACTTGCAGAATCCAGTGAATCATCAAAACTTTGAACGCACCTTGCGCTTCCGGGTTACTCCTGGGAGCATGCTTGTCGGAGTGTCTGTTGACACCACTCGCCCCCCGCCCCCTCTCCTCCCTCGGGAGAGAGGGGTCGCGTGGGCGGACTCTGAGTGTTCCGGAGCATCCCACGCTCCGAGTGCACCAAATCTCGTGAACGAAGCCTCTCGCG-CCCTGCCGCACAAGAGTTGTTGACGGCGTTCGCTTCGGCGGCGACTCTCGACTCACGAAACGTGCGCGCAGAGCCGCGGGCTTCTTCCGGCGCTCCAGAGAAGAACTGGAATCCGTACCACTTT

>LesB [organism=Lessonia spicata] [country=Chile: Castillo channel, Torpedo Island, Katalalixar National Reserve] internal transcribed spacer 1, partial sequence

ACCCCCCCGCTCTACAAATTGTCTGTGACGT-CGCCGTGGAAACTCCCTCGGAGGCGAGCGA-GCGATCGTCTAA---ACCCCGAGAAAGTGAAGCCGTTATGCGAAGTTGGGCGAGGGGCGCCTCGCCGAGGGTTTTTCTTTTCCCTTTCCCCTTCTTGTCTC-TTTTCTTCCCTTTTCGGGGATTTACGGGACGGGACGGGAAGGGTGAAGGAAGCTCTCGAACCAAAGCGCACCCCACTTTTCAACCCCATTAAACTCTGAATCTGAACTC-AAAGGGGGGCAGCGCTGTGCCGCGGCTCCCCCAACCTTTAACGTTGTAAAACTTTCAGCGACGGATGTCTTGGCTCCCACAACGATGAAGAACGCAGCGAAATGCGATACGTCTTGCGACTTGCAGAATCCAGTGAATCATCAAAACTTTGAACGCACCTTGCGCTTCCGGGTTACTCCTGGGAGCATGCTTGTCGGAGTGTCTGTTGACACCACTCGCCCCCCGCCCCCTCTCCTCCCTCGGGAGAGAGGGGTCGCGTGGGCGGACTCTGAGTGTTCCGGAGCATCCCACGCTCCGAGTGCACCAAATCTCGTGAACGAAGCCTCTCGCG-CCCTGCCGCACAAGAGTTGTTGACGGCGTTCGCTTCGGCGGCGACTCTCGACTCACGAAACGTGCGCGCAGAGCCGCGGGCTTCTTCCGGCGCTCCAGAGAAGAACTGGAATCCGTACCACTTT

>LesC [organism=Lessonia spicata] [country=Chile: Castillo channel, Torpedo Island, Katalalixar National Reserve] internal transcribed spacer 1, partial sequence

ACCCCCCCGCTCTACAAATTGTCTGTGACGT-CGCCGTGGAAACTCCCTCGGAGGCGAGCGA-GCGATCGTCTAA---ACCCCGAGAAAGTGAAGCCGTTATGCGAAGTTGGGCGAGGGGCGCCTCGCCGAGGGTTTTTCTTTTCCCTTTCCCCTTCTTGTCTC-TTTTCTTCCCTTTTCGGGGATTTACGGGACGGGACGGGAAGGGTGAAGGAAGCTCTCGAACCAAAGCGCACCCCACTTTTCAACCCCATTAAACTCTGAATCTGAACTC-AAAGGGGGGCAGCGCTGTGCCGCGGCTCCCCCAACCTTTAACGTTGTAAAACTTTCAGCGACGGATGTCTTGGCTCCCACAACGATGAAGAACGCAGCGAAATGCGATACGTCTTGCGACTTGCAGAATCCAGTGAATCATCAAAACTTTGAACGCACCTTGCGCTTCCGGGTTACTCCTGGGAGCATGCTTGTCGGAGTGTCTGTTGACACCACTCGCCCCCCGCCCCCTCTCCTCCCTCGGGAGAGAGGGGTCGCGTGGGCGGACTCTGAGTGTTCCGGAGCATCCCACGCTCCGAGTGCACCAAATCTCGTGAACGAAGCCTCTCGCG-CCCTGCCGCACAAGAGTTGTTGACGGCGTTCGCTTCGGCGGCGACTCTCGACTCACGAAACGTGCGCGCAGAGCCGCGGGCTTCTTCCGGCGCTCCAGAGAAGAACTGGAATCCGTACCACTTT
